# Health behaviours and lifestyle challenges among school children: A qualitative research from Rajasthan, India

**DOI:** 10.1371/journal.pone.0351408

**Published:** 2026-07-07

**Authors:** Mukti Khetan, Purva Paliwal, Rupal Sharma, Monika Meghwal, Tinku Yogi, Ramesh Kumar Sangwan, Bontha V. Babu

**Affiliations:** ICMR-National Institute of Health Research, Jodhpur, India; Faculty of Medicine, Parul Institute of Public Health, Parul University, INDIA

## Abstract

**Background:**

Non-communicable diseases (NCDs) account for 74% of global deaths, significantly affecting children and adolescents, particularly in low and middle-income countries. Addressing risk factors such as smoking, poor diet and physical inactivity through school-based interventions is crucial for reducing NCD prevalence and improving long-term health outcomes among youth. Therefore, this study aims to explore school children’s lifestyle habits, identify barriers and assess facilitators for adopting a healthy lifestyle.

**Methods:**

This study utilized qualitative research methodology (focus group discussions (FGDs) and in-depth interviews (IDIs)) to collect comprehensive insights from school children, teachers, parents, and school canteen staff. It was carried out in both rural and urban regions of Jodhpur district, Rajasthan, a western state of India with a purposive sample of 31 participants. Thematic analysis was performed using NVivo 14.

**Results:**

Our study reveals that while participants have a basic understanding of NCDs, there is a significant gap in their knowledge of NCD-related health initiatives. Barriers to healthy lifestyles include limited access to recreational spaces, unhealthy food options, and socioeconomic factors. However, family and peer support and school initiatives are crucial in promoting healthy behaviours.

**Conclusion:**

Bridging the gap between health knowledge and practice requires a holistic approach. Enhancing communication about health programs, improving access to recreational spaces, and implementing policies to regulate unhealthy foods are essential. Engaging families and educators and integrating health education into school curriculums will help promote healthier behaviours and reduce the risk of non-communicable diseases.

## Introduction

Non-communicable diseases (NCDs) pose a significant global health challenge, accounting for 74% of total deaths globally, substantially impacting individuals in their productive years [1]. Children and adolescents, numbering more than 2.1 billion globally, are increasingly affected by NCDs, including cardiovascular diseases, cancers, chronic respiratory disorders, diabetes, mental health issues, and injuries, predominantly in low- and middle-income countries [2]. Tobacco-related deaths alone contribute to over 8 million fatalities annually [3]. At the same time, approximately 830,000 deaths each year can be associated with insufficient physical activity [4].

Adolescence is a critical period in which health related behaviors such as smoking, alcohol use, and physical inactivity are formed, which often persist into adulthood, significantly impacting long-term health outcomes including NCDs [5]. Socio-economic factors significantly influence adolescents’ dietary habits and physical activity levels and play a pivotal role in determining their health outcomes [6,7]. Enhancing nutritional knowledge and promoting healthier lifestyles are essential in combating the rising prevalence of NCDs among children and youth [8,9].

Indian studies have documented rising trends of unhealthy dietary practices, limited physical activity, and early initiation of tobacco use among adolescents, influenced not only by family and peers but also by school policies and community environments [10–12]. Qualitative research from diverse regions of India, including Odisha, Maharashtra, and West Bengal, reveals persistent gaps in awareness and practice, as well as challenges posed by social norms, infrastructural shortcomings, and inconsistent health messaging in schools [13,14]. Importantly, these studies underscore the unique barriers faced by students in resource-limited, rural, and culturally diverse settings-gaps particularly pronounced in western states like Rajasthan.

Schools have been recognised as key platform for implementing interventions to mitigate NCD risk factors among children and adolescents. Initiatives like the Diabetes Awareness and Prevention Education program have successfully raised awareness and promoted healthier lifestyles among students [15,16]. However, sustaining these efforts and evaluating their long-term efficacy remain ongoing challenges [16]. Evidence from recent school-based interventions indicates that success depends upon the engagement not just of students, but also teachers, parents, and crucially, canteen staff who shape the everyday food environment [17].

Early intervention targeting these risk factors is crucial for reducing premature mortality and improving global public health [18]. Environmental factors, including access to nutritious food and health literacy, further shape adolescent health behaviors and require targeted interventions for effective NCD prevention [19,20]. Recent qualitative studies further call for research that captures segmented, contextually specific challenges, especially in less-studied regions.

In Rajasthan, the burden of NCD risk factors among school children is notably high, with significant proportions being overweight or obese and engaging in tobacco consumption

[10,21]. Recent regional studies also document disparities between rural and urban populations and highlight the need for interventions tailored to local socio-economic and cultural realities [10,21]. Despite national policies and campaigns, few qualitative research has explored how students, parents, teachers, and non-teaching staff perceive and experience these barriers and facilitators in western India, especially in Rajasthan.

Understanding and addressing behavioural risk factors, such as diet, physical activity, and tobacco use among school children, can pave the way for effective primary prevention strategies against NCDs. In this context, this study from Rajasthan, India provides comprehensive insights into school children’s lifestyle habits in both rural and urban areas along with identification of barriers and assessing facilitators for adopting a healthy lifestyle from perspective of students, teachers, parents and canteen staff.

## Methods

This study is part of implementation research towards primordial prevention of NCDs among school-going children.

### Study design

This paper is based on data collected with a qualitative approach utilizing focus group discussions (FGDs) and in-depth interviews (IDIs). This methodology enables the collection of detailed and comprehensive data on the experiences and perspectives of school children, teachers, parents, and canteen staff [22]. In total, 2 FGDs and 15 IDIs were conducted, with the distribution as follows: rural government - 1 FGD, 6 IDIs; urban government - 1 FGD, 2 IDIs; rural private - 3 IDIs; and urban private - 3 IDIs.

### Study site and participants

The study was conducted in rural and urban areas, spanning government and private schools in Jodhpur district of Rajasthan, a western state of India. The participants include key stakeholders – students, teachers, parents and canteen staff. The school authorities were contacted first, and after institutional permission, participants were invited by research team based on informed written consent of participants who volunteered, which includes – students specifically from grades 7, 8, and 9, their schoolteachers, parents, and school canteen staff members from the study area. This varied group of participants provided information and an understanding of the factors influencing health behaviours within school settings.

### Sample size

The total sample size is thirty-one, and was selected purposively. The sample included eighteen schoolchildren, six teachers, five parents, and two canteen staff members from rural and urban government schools and private schools.

#### Data collection and management.

We have utilised two data collection methods: FGDs and IDIs. The FGDs and IDIs used interview guides to extract detailed information about the research questions. The checklist of FGD and IDI had three main sections -i) understanding of NCDs, their risk factors and related programs, ii) challenges, barriers and facilitators of healthy lifestyle as well as healthy diet, physical activity and no tobacco use, iii) support and suggestions for healthy behaviours. Students, teachers and parents were invited to participate in the study voluntarily. Participants were briefed about the study and provided with printed materials to ensure comprehension. Informed written consent was obtained from all participants after providing a briefing about the project, with consent forms provided in their preferred language (Hindi or English). Consent forms were secured from teachers, canteen staff, and parents, while assent forms were signed by parents/guardians and teachers on behalf of the students. Interviews were conducted at the schools during the month of 06 April and 29 May 2024. Participant identities were anonymised using unique identifiers. Interviews were conducted by research team members in a private space provided by the school. The IDIs were conducted with stakeholders to accommodate individual availability, busy schedules, to get depth on personal experiences and due to possible sensitivity of some topics. Whereas, the FGDs were conducted with students specifically to encourage interaction and active involvement of students, explore share norms, peer influences, and perceived barriers and facilitators to healthy lifestyle. The FGD and IDI checklists were developed through literature review and brainstorming among researchers. These checklists were developed in English and later translated into Hindi, the local language. The FGDs and IDIs were conducted by the research staff, who were trained in Qualitative research. The average duration of each IDI was 35-40 minutes, and the FGD took around an hour and a half. All FGDs/ IDIs were audio recorded with consent, in addition to taking notes. One student FGD was conducted in an urban government school and another in a rural government school, capturing diversity across public school contexts. No FGDs were held in private schools due to the limited availability of students and parents. Instead, we conducted IDIs with teachers and parents in both urban and rural private schools, ensuring representation across public–private and urban–rural settings. Only two canteen staff members from eight schools were available and consented to participate, so in-depth interviews were conducted with both. The data saturation was defined as the point at which responses were repeated, no new point or important insights related to the healthy lifestyle behaviours among adolescents emerged. A simple saturation grid was maintained based on key topics of barriers and facilitators to a healthy lifestyle. Saturation of main categories was first observed after the fourth IDI, when no new codes were identified among the responses from parents and teachers, while among students’ saturation was observed after 2 FGDs and 1 IDI. Additional IDIs were conducted to verify the saturation, which generated only repetitions of existing themes. On this basis, it was concluded that data saturation had been reached and it’s unlikely that new insights could be added. FGDs and IDIs audio recordings were transcribed, translated, and stored in password-encrypted files to ensure data security and confidentiality.

#### Data analysis.

The audio of FGDs and IDIs was transcribed into Hindi, in which they were conducted. Notes were used as an adjunct while transcribing. These transcribed scripts were translated into English. Standard methodology was followed during transcription and translation. Thematic analysis was conducted using NVivo 14, employing both inductive and deductive approaches while following standard procedures [23]. Stratified thematic analysis for each stakeholder group-students, parents, teachers, and canteen staff was used. Data from each group were first analyzed separately to identify themes specific to their experiences, followed by a cross-group comparative synthesis to highlight areas of convergence and divergence in thematic patterns.

### Ethics approval

All procedures involving human participants were conducted in accordance with the ethical standards of the Institutional Ethics Committee of the National Institute for Implementation Research on Non-Communicable Diseases, Jodhpur, India, and in compliance with applicable local legislation and institutional requirements. Ethical approval for the study was obtained on 26 December 2023 (Approval No. IEC-NIIRNCD/2023/DEC/FR/007). No potentially identifiable participant images or personal data are presented in this manuscript.

### Consent to participate

Written informed consent was obtained from all relevant participants, including parents and teachers, prior to enrolment in the study. For student participants, written informed consent was obtained from parents or legal guardians, as appropriate, before participation.

## Results

This section presents the outcomes of the investigation. It commences by delineating the characteristics and socio-demographic profiles of the study participants, as summarized in Table 1.

**Table 1:** Details of the participants.

Participant(s) (n = 31)	Code	No. of participants	Type of interview	Gender distribution	Age	Experience	Occupation	Location	School Type
Students Group 1	S1 to S8	8	FGD	5 Male, 3 Female	11-14	–	Student	Urban	Government
Students Group 2	S9 to S16	8	FGD	4 Male, 4 Female	11-15	–	Student	Rural	Government
Student 3	S17	1	IDI	Male	15	–	Student	Rural	Government
Student 4	S18	1	IDI	Male	13	–	Student	Rural	Government
Teacher 1	T1	1	IDI	Male	39	2 years	Teacher	Urban	Government
Teacher 2	T2	1	IDI	Male	46	23 years	Teacher	Urban	Government
Teacher 3	T3	1	IDI	Female	29	9 years	Teacher	Urban	Private
Teacher 4	T4	1	IDI	Male	32	16 years	Teacher	Rural	Private
Teacher 5	T5	1	IDI	Female	43	23 years	Teacher	Urban	Private
Teacher 6	T6	1	IDI	Female	40	10 years	Teacher	Urban	Private
Parent 1	P1	1	IDI	Male	40	25 years	Farmer	Rural	Government
Parent 2	P2	1	IDI	Male	47	20 years	Admin Staff	Rural	Private
Parent 3	P3	1	IDI	Male	40	15 years	Teacher	Rural	Private
Parent 4	P4	1	IDI	Female	49	18 years	Teacher	Rural	Government
Parent 5	P5	1	IDI	Female	39	17 years	Admin Staff	Urban	Private
Canteen Staff	CS1	1	IDI	Female	26	2 months	Canteen staff	Rural	Government
Canteen Staff	CS2	1	IDI	Female	28	1 year	Canteen staff	Rural	Government

By thematic analysis, we identified key themes and subthemes, which are presented in Table 2.

**Table 2:** Themes and subthemes.

	Theme	Subtheme	Key Conclusion
1.	Awareness, knowledge & practices of healthy lifestyle	1.1. Awareness of NCDs and NCD-Related Programs	• Basic understanding of NCDs with few students and teachers • Incomplete knowledge of risk factors among all participants • Limited awareness of government health initiatives
		1.2. Knowledge of healthy lifestyle behaviors	• Maintaining daily routine with morning exercise/yoga is essential • Acknowledged balanced diet with high fruits and vegetables • Aware of harmful effects of tobacco • Shared personal stories on tobacco use and its economic and health impact
		1.3. Practices of healthy lifestyle behaviors	• Admitted to occasional eating of fast food • Bring lunch boxes with packaged & fast food • Busy schedules and appealing taste increase reluctantly to healthy food • Insufficient organized physical activity
2.	Challenges/barriers to healthy lifestyles	2.1. Accessibility barriers	• Inadequate recreational spaces • Shortage of physical education instructors • Time constraints and limited resources
		2.2. Behavioral and practical barriers	• Taste preference and time constraint as barrier • Impact of commercial advertisements and social media • Familial influence • Difficulty in sustaining healthy habits
		2.3. Social and economic barriers	• Economic and cultural influence • Peer and parental influence • Gender specific physical activity barrier
		2.4. Parental attitudes and practices	• Convenience and time constraints • Parents as role model and resistance to dietary changes • Cultural and habitual food preferences
3.	Facilitators	3.1. Family and peer support/influence	• Family support/motivation and bonding • Striving to balance preferences and nutrition • Peer influence/ sharing gives exposure to new food
		3.2. School initiatives	• Policies against tobacco use in school • Incorporating physical activity in curriculum • Mid-day meal program/fruit break with lunch breaks
		3.3. Health education and awareness programs	• Need of health education in curriculum • Technology’s dual impact in education
4.	Recommendations	4.1. Future initiatives and program development	• Teachers emphasize on consistent monitoring of health diet • Organizing sports events/programs • Parents support positive habits
		4.2. Parental and community support	• Community parks and gyms to encourage physical activity • Parental involvement and creative approach to diet
		4.3. Policy and environmental changes	• Strict health policies for regular health check • Community involvement and food regulations
		4.4. Enhancing school environment on healthy lifestyle	• Adequate sports facilities with variety of organized physical activities • Address stress and mental well-being • Emphasizing cleanliness and hygiene

### Theme 1: Awareness, knowledge and practices of healthy lifestyle

#### Awareness of NCDs and NCD-related programs.

Participants showed a basic understanding of NCDs, but only a few teachers and students could differentiate between communicable and non-communicable diseases. Knowledge about the risk factors for NCDs and the transmission modes of communicable diseases was limited among parents, students, and some teachers. Awareness of the Ayushman Bharat program was limited to a few parents and teachers, and none were familiar with the Rashtriya Bal Swasthya Karyakram (RBSK) program and the National Programme for Prevention and Control of Non-communicable Diseases (NP-NCD) or other related health initiatives. Nevertheless, most participants knew of specific health benefits associated with government schemes and campaigns, including distributing vitamin D and iron folic acid pills and programs like Fit India Day and Run for India.

*“Communicable, cough is spreading. A disease that does not spread is called non- communicable, and a disease that spreads is called communicable. Communicable diseases include malaria and dengue fever. As with non-communicable skin problems. Have skin problems”- S9 (Rural Government School Student)*

*“Ayushman Bharat, I’m familiar with this but RBSK, I’m not familiar with it “ – T4*

*(Rural Private School Teacher)*

*“Apart from Ayushman Bharat, we have a lot of events, there was Fit India Day, Run for India” - T2 (Urban Government School Teacher)*

#### Knowledge of healthy lifestyle behaviors.

Participants, including teachers, students, and parents, recognized regular exercise and maintaining a structured daily routine as essential for a healthy lifestyle. Teachers stressed the importance of incorporating physical activities and sports, while some students mentioned morning exercises such as cycling and Surya Namaskar (Sun Salutation, a yoga posture). Parents highlighted physical activity as vital for overall health and disease prevention. Additionally, the significance of a balanced diet, rich in fruits and vegetables, was acknowledged. Recommendations included having substantial lunches and lighter dinners for better health and avoiding oily and fried foods, particularly in hot weather. Teachers provided education on the risks of tobacco use, though only some schools included this in their curriculum. Both students and parents were aware of tobacco’s harmful effects. They shared personal anecdotes about its economic and health impacts, with parents and teachers advising children to avoid such unhealthy habits.

*“In a healthy life, first look at the lifestyle; you should have routine work with nature... there should also be time for physical exercise and sports” -T2 (Urban Government School Teacher).*

*“One should wake up early in the morning, take a bath, and get ready before sunrise”*

*-S10 (Rural Government School Student).*

*“There are chapters on tobacco... the child also understands how dangerous it is” - T5 (Rural Private School Teacher)*

#### Practices of healthy lifestyle behaviors.

Most students reported a preference for eating at home but admitted to occasionally indulging in junk food, such as pani-puri (street food consisting of small, crispy, hollow fried dough balls filled with a spicy, tangy water, along with a mixture of mashed potatoes, chickpeas, tamarind chutney, and spices), samosas (a triangular or cone-shaped pastry filled with a savory mixture, typically spiced potatoes, peas, etc.), and chowmein (Chinese dish made of stir fried noodles mixed with vegetables, and sometimes meat like chicken), due to personal taste preferences. Teachers noted that students often bring packaged or fast foods, such as Maggie, noodles, and bread, or purchase snacks like biscuits and chips in their lunch boxes. This tendency might be linked to parents’ busy schedules, which lead to quicker meal preparation. Parents observed that junk food’s appealing taste often outweighs that of vegetables, leading to children’s reluctance to eat healthier foods. In response, parents try creatively incorporating vegetables, such as stuffed parathas and cutlets. Despite these difficulties, all groups-parents, teachers, and students-recognized the value of maintaining a healthy diet. Some students mentioned frequent gym visits to achieve a fit physique, inspired by role models. Teachers reported that only some students engaged in organized physical activities during school hours, while parents enrolled their children in sports leagues and engaged in activities like playdates or bicycle rides to encourage physical exercise.

“*If I am able to get some time, I like to dance. This helps me to do some physical activity” -* S1 *(Urban area Government School Student).*

“*like chowmein or a sandwich is not good for us, we are not able to stop it. We have to eat.”-* S13 *(Rural area, Government School)*

“*It has become a routine for children. They want fast food once a week. They know that it has fat in it. But they still eat it”-* S3 *(Urban area, Government School)*

### Theme 2: Challenges/barriers to healthy lifestyles

#### Accessibility barriers.

A significant challenge in maintaining a healthy lifestyle is the inadequate availability of recreational spaces, such as parks, playgrounds and amusement areas. Students expressed dissatisfaction with the limited space for physical activities at home. Additionally, the lack of physical education instructors and the high number of students per class were identified as obstacles to participating in physical activities. Both teachers and students of urban and private schools highlighted the shortage of parks and open spaces for exercise, pointing to a significant infrastructural gap. Teachers and parents also noted the lack of designated time for physical activities during school hours, sometimes leading to neglect of structured exercise. Students from urban as well as rural area schools mentioned that academic demands and household chores further restrict their ability to engage in physical activities. For students time constraints and limited resources compound these barriers. Additionally, the easy availability of tobacco and junk food in and around school environments serves as a barrier to maintaining a healthy diet and avoiding tobacco use.

*“There are no parks in our neighbourhood for outdoor activities” -T6 (Rural Private School Teacher).*

“*Whenever I go to the ground, sometimes sir (Physical education instructor) does not come and sometimes he does not take us at all” -* S10 *(Rural Government School Student).*

*“There is no space, there are only lanes and small roads in front of the house.” - S8 (Urban Government School)*

#### Behavioural and practical barriers.

A key behavioural challenge is the difficulty in adopting and maintaining healthy habits, which is evident in students’ and their families’ behaviours and attitudes. Teachers from both rural and urban areas observe that while students may recognise the importance of a healthy diet, they often find it challenging to consistently follow through and sustain these habits. Students from all type of schools admitted to struggles reconciling their knowledge with actions that cite internal conflicts as a significant barrier. Familial influences, such as parental tobacco use and limited awareness of healthy eating practices compound this issue. Additionally, practical barriers exist, as teachers face difficulties in allocating adequate time for physical education within the school schedule, which hinders the promotion of regular physical activity.

*“The difficulties are that students listen to you but does not follow you”- T2 (Urban Government School Teacher).*

*“My mind tells me to drink (Sting energy drink) but my inner conscience says not to drink, it will cause harm” -S17 (Rural Government School Student).*

*“In 6 days, we get the 4 periods for physical education. So, 48 periods are there. In 48 periods, only 4 periods are there for games. So, you can see that the margin is quite low. And it should be enhanced”- T3 (Urban Government School Teacher).*

Taste preferences are a notable practical barrier to healthy eating among students. Both teachers and students point out that taste plays a crucial role in dietary choices, with teachers struggling to encourage nutritious foods that may not be as appealing. Students acknowledged a preference for junk food despite understanding its negative health impacts. The perceived blandness of healthier options often enhances the attractiveness of unhealthy junk foods.

The influence of commercial advertisements and social media is also a significant issue. Students report being influenced by advertisements that present certain foods or behaviors as

desirable. At the same time, teachers note that misleading information on social media can distort children’s understanding of health and nutrition. Students also mention challenges in accessing fresh vegetables, the convenience of unhealthy snacks, and the wide availability of such options. Additionally, the time required to prepare healthy meals poses a practical difficulty that further obstructs the adoption of healthy eating habits.

*“Junk food like pizza and burgers are tasty. They are not good for us. But we like its taste. That’s why we eat it” - S7 (Urban Government School Student).*

*“We don’t like to eat green vegetables like spinach or broccoli. We leave it after eating fast food “- S3 (Urban Government School Student).*

#### Social and economic barriers.

Economic factors such as the expense of nutritious foods and physical activities are significant barriers to maintaining a healthy lifestyle, as noted by both teachers and students. Teachers understand families’ financial difficulties and suggest that better awareness and resourcefulness might help make healthy eating more accessible. Although economic challenges persist, limiting access to more nutritious options, parents often meet their children’s requests for packaged or junk food due to financial constraints. These economic limitations also affect children’s ability to access nutritious foods and participate in physical activities or social events without sufficient support. The emphasises on economic constraints and food affordability was more by rural participants.

Cultural practices also play a role in shaping behaviors and can obstruct healthier living. Social norms that encourage dining out and consuming unhealthy foods during social gatherings can make it challenging to adopt healthier eating habits. In rural areas, there is often limited knowledge about diverse and nutritious foods. Canteen staff reported providing a variety of foods in midday meals, but the inclusion of green leafy vegetables in village schools and households remains minimal.

*“You can feed yourself very good things even at a cheap price...You should know how*

*to utilize available resources“ - T5 (Rural Private School Teacher).*

*“Sometimes when papa doesn’t have money and can’t bring it...so can’t bring/buy”- S13 (Rural Government School Student).*

Teachers note that gender-specific cultural practices affect physical activity, limiting girl children’s opportunities to participate in diverse and inclusive sports. Parents also mentioned that their girl child does not prefer outdoor games and recreational activities due to insecurities and introversion.

*“It has become a culture. When guests come to our house, we discuss about it...We go together. We spend time together. We have lunch together. They don’t think that it is unhealthy” -S5 (Urban Government School Student).*

*“The environment at home is that if there is cricket, only boys will play this game, if there is badminton, only girls will play”- T6 (Rural Private School Teacher).*

Peer pressure and social norms around tobacco use illustrate the social pressure to engage in unhealthy behaviours. Teachers from both rural and urban schools highlighted social media’s role in this issue, often amplifying negative influences despite its potential for positive learning. One teacher’s anecdote about a student offering drug in class underscores the pervasive and normalised nature of tobacco abuse among peers.

*“Sometimes my friend takes ‘amal’(opium). He tells me to take ‘amal’ too. I said no I don’t. It harms our body” - S17 (Rural Government School Student)*

#### Parental attitudes and practices.

A significant challenge is the influence of convenience and time constraints on parents’ food choices for their children. Teachers have observed that busy parents often opt for quick, convenient options such as pizza and burgers for school lunches, placing ease of preparation over nutritional value. Cultural and habitual practices also play a role, with some parents preferring traditional ‘desi’ foods, even if they are less healthy, over more nutritious alternatives. The impact of modern influences, like television and mobile phones, further undermines traditional healthy eating habits, with media and sedentary behaviours contributing to the problem.

Teachers have noticed that resistance from parents to altering their children’s diets can be a barrier to healthier lunch options. Some parents show reluctance to change and react negatively to dietary recommendations that indicate a preference for convenience due to their busy lifestyles. Additionally, parental behaviours, such as tobacco use, insufficient physical activity, and dietary choices, set examples for their children, influencing their health practices and attitudes.

*“Mothers send pizza, burger in tiffin. They are in a hurry; they prepare pizza burger in tiffin and sends it”- T5 (Rural Private School Teacher).*

*“There is no such challenge. I mean our food habits are always desi” - P1 (Parent, Rural Government School)*

*“If parents also eat tobacco, then what will the children do”- T5 (Rural Private School Teacher).*

### Theme 3: Facilitators

#### Family and peer support/influence.

Family support is essential in motivating children to adopt healthy behaviors. Students frequently mention that family encouragement is pivotal in guiding their daily routines and academic efforts. Parents work to balance catering to their children’s preferences while promoting healthy eating habits, ensuring their children receive proper nutrition while accommodating their tastes. Engaging in after-school activities and household chores also allows parents to stay physically active and strengthen family bonds.

Peer influence significantly affects children’s behaviours as well. Observations from teachers, parents, and students of the urban as well as rural areas highlight that peer groups shape children’s habits, including their food choices, sports involvement, and attitudes toward tobacco. Students report that sharing meals at school with friends exposes them to different foods, which can spark an increased interest in consuming vegetables and fruits.

*“My father encourages me; my brother encourages me and my mother too. They say that you should study only then you will get a job” - S17 (Rural Government School Student).*

*“He doesn’t eat brinjal, he doesn’t eat onion and he doesn’t eat anything else, so sometimes we make parathas for him and give it to him” -P1 (Parent, Rural Government School).*

*“A positive pupil influence, a hard-working companion, then 100% those things will affect you. Suppose today I am bringing vegetables, fruits, and something like this in my tiffin... I accepted whatever I saw, ma’am this is a fact and ma’am this is my own experience that whatever your friend circle is, your future will be like that”- T6 (Rural Private School Teacher).*

“*I feel that 80% of the children get affected by looking at each other, I have felt this thing here.” T6 (Rural Private School Teacher)*

#### School initiatives.

School initiatives are essential for promoting students’ mental and emotional well-being. Three schools have implemented strict policies to prevent tobacco use on campus, supported by regular inspections to ensure compliance. These measures highlight the schools’ commitment to maintaining a safe and health-conscious student environment. Additionally, many schools incorporate physical activities into their curricula and dedicate time during prayer or assembly for fitness routines.

Government schools support healthy eating by providing meals through mid-day meal programs. In contrast, private urban schools emphasise proper eating habits and hygiene education, encouraging students to bring nutritious meals from home. For instance, one school requires students to bring fruits for a 10-minute fruit break, separate from recess, to ensure they receive necessary nutrients throughout the day. Furthermore, schools enhance community and cultural awareness through various celebrations and competitions, fostering creativity and social interaction and strengthening the school community.

*“The use of tobacco is prohibited in the school premises, and children do not use it. Whether it’s students or teachers, it is illegal here. There is no wet canteen here for this reason, and even polythene use is banned in this school” - T1 (Urban Government School Teacher).*

*“It means when we, like parents-teachers meeting, all parents are informed about food, what food should be given to children and what should not be given” -T1 (Urban Government School Teacher).*

“*We are having a short break also, that is known as a fruit break for 10 minutes, which is playing a very important role” T4 (Rural, Private school)*

#### Health education and awareness programs.

Teachers stress the importance of including health education in the school curriculum, ensuring that it is integral to students’ learning experiences. This structured approach incorporates health awareness into the academic framework, reinforcing its importance alongside other subjects. Students’ feedback from both rural and urban schools was consistent, indicating that external factors, such as media advertisements and public health messages, also shape their understanding of health. Media warnings about unhealthy behaviours serve as ongoing reminders, complementing the lessons received in school.

The role of technology in health education is complex. Some parents believe that technology, such as smartphones and televisions, negatively impacts their child’s lifestyle by increasing sedentary behaviour. On the other hand, other parents view technology as a beneficial educational resource, offering an alternative to traditional tuition centres and allowing working parents to maintain oversight of their child’s learning.

*“In the curriculum... Every student’s curriculum includes information about health” - T1 (Urban Government School Teacher).*

*“First it is written in the advertisement – ‘Alcohol is injurious to health’. In this film, it is also told when someone is seen drinking”-S10 (Rural Government School Student).*

*“Mobile is bad and good for us but we will get good by watching good things and bad if we watch worse things. Our TV is also like this ma’am.” -S13 (Rural Government School Student).*

### Theme 4: Recommendations

#### Future initiatives and program development.

Teachers emphasise the need for consistent monitoring of students’ health behaviours and the involvement of parents in supporting positive habits. Regular inspections of students’ meals help ensure they consume nutritious food, which is vital for their health and academic performance. Additionally, the importance of developing inclusive sports programs is highlighted. These programs allow all students to engage in physical activities, promoting fitness, motivation, and inclusivity. Schools can significantly enhance students’ physical health and social skills by organizing sports events and tournaments, encouraging a lifelong commitment to physical activity.

*“To improve their health, one thing is that their tiffin should be surveyed daily or teachers should check it and parents should support it” – T2 (Urban Government School Teacher).*

*“The second thing that can be corrected is that the tournament should be organized at such a level that every child gets a chance to participate in it. So, the child gets a motivation”- T2 (Urban Government School Teacher).*

Teachers especially from rural area highlighted the need for regular health screenings and the involvement of specialized teams to monitor and educate students about health risks. They advocate for periodic evaluations by healthcare professionals to promptly detect and address any health issues. Teachers from both government and private schools also believe that integrating health education from an early age lays a strong foundation of health knowledge, which evolves as students’ progress in their education, helping them make informed decisions about their health.

*“There should be a team for these policies that comes to the school every six months or three months and does observation” -T5 (Rural Private School Teacher).*

*“There should be a team that comes and checks every month. Check all these things, yes, this because Ma’am, it is not like that if we are using tobacco and no one will come to know, that thing is not there, there should be a team of doctors who check all the students of the class every month” – T6 (Rural Private School Teacher).*

#### Parental and community support.

Teachers suggest each community should have parks and gyms to encourage physical activity. Such accessible recreational spaces would enable children and adults to exercise, promoting a

fitness culture within the community. Transforming school grounds into community parks could extend the health benefits of schools beyond regular hours. Moreover, teachers and parents from both private and government schools agree on the importance of parental involvement in developing healthy eating habits at home. Informed parents are more likely to make nutritious food choices for their children, which supports overall health and prevents lifestyle-related diseases. Teachers also recommend creative methods to make healthy foods more appealing, such as incorporating vegetables into familiar and enjoyable dishes, to encourage children to consume nutritious foods more willingly.

*“Every colony should have its own park in which an open gym is open... The school grounds should also become a park and it should also become a part of the school” – T2 (Urban Government School Teacher).*

*“Parents should be educated on the importance of a healthy diet and lifestyle” – T5 (Rural Private School Teacher).*

#### Policy and environmental changes.

Teachers propose offering a variety of physical activities to engage students with different interests and abilities. Recognizing that traditional sports may not appeal to everyone, alternatives like dance can effectively encourage physical activity among all students, including those who might not participate in conventional sports. Teachers also emphasized the importance of enforcing strict health policies within schools. Comprehensive guidelines, including regulations on food offerings, mandatory physical education, and regular health assessments, need to be implemented rigorously to ensure a consistently healthy environment. Additionally, students have highlighted the value of strong policies and suggested that local shopkeepers should be involved in raising awareness about the harmful effects of tobacco.

*“First, ask their choice whether you will play Kabaddi, you will play football or any other game... If someone doesn’t play games then he should go dancing. Dancing is the best activity for fitness” – T2 (Urban Government School Teacher).*

*“Policymakers need to enforce health guidelines strictly in schools” – T5 (Rural Private School Teacher).*

*“The shopkeeper should explain that Gutka should not be taken, which will improve people’s health… TV also tells us that one should not smoke. Before the picture restarts, it is told that we should not smoke. Yet everyone does it “- S9 (Rural Government School Student).*

#### Enhancing school environment on healthy lifestyle.

Teachers from both urban and government schools emphasised the need for schools to be well-equipped to support students’ physical health and activities. This includes having adequate sports facilities and trained staff to encourage active participation. Sufficient space for physical activities is also essential. Teachers also recognise the importance of motivating students by offering various exercise options, such as traditional sports, yoga, and even household chores that contribute to physical fitness.

One teacher proposed implementing structured dietary plans to help students develop healthy eating habits and establish a routine, fostering responsibility for their dietary choices. Teachers also emphasise the importance of cleanliness, involving students in keeping the school environment clean. Additionally, a teacher from an urban setting suggested addressing students’ stress to support their mental and emotional well-being, acknowledging the link between these aspects and overall health.

*“Provide them with the required material, provide them with the required time, resources, ground and sports items, keep trained coaching, keep them alert all the time” – T2 (Urban Government School Teacher).*

*“We should at least be given a period for sports in school. Playing is very important for health” – S9 (Rural Government School Student).*

*“In our school, there is a class or even during prayer, where the science teacher explains*

*what should be included in their diet“- T1 (Urban Government School Teacher).*

By incorporating it into the curriculum and daily activities, teachers are pivotal in providing health education. Their role in balancing discipline with support helps create an environment that fosters physical fitness and overall health. Teachers need adequate resources and training to lead health education and wellness programs. These efforts are most impactful when supported by both parents and the community. A teacher’s feedback emphasizes the importance of investing in resources and ongoing professional development to keep teachers updated with current health education practices. Institutional policies also play a crucial role in supporting health education. Policymakers should enforce health guidelines and ensure schools have the proper facilities and clean environments to support these educational activities. Parental involvement can reinforce healthy habits at home, while community support through health awareness initiatives and necessary infrastructure can further strengthen teachers’ efforts.

*“Parents should be educated on the importance of a healthy diet and lifestyle” – T5 (Rural Private School Teacher).*

*“Provide them with the required material, the required time, resources, ground and sports items, keep trained coaching, keep them alert all the time” – T2 (Urban Government School Teacher).*

“*Policymakers need to* *enforce* *health guidelines strictly in schools” – T5 (Rural Private School Teacher).*

## Discussion

India has launched several initiatives to prevent and manage NCDs, including the National Multisectoral Framework (2017–2022), the National Monitoring Framework with specific targets and indicators, and the National Program for Prevention and Control of Cancer, Diabetes, Cardiovascular Diseases, and Stroke (NPNCDCS) [23]. Ayushman Bharat includes a School Health and Wellness Programme (AB-SHWP), which aims to improve the health and well-being of school children. This initiative focuses on health promotion and disease prevention through various activities in schools. RBSK focuses on early screening and intervention for children’s health conditions. Despite their potential, our study showed that awareness of these programs remains limited. Participants under the study have a basic understanding of NCDs and communicable diseases. However, there is a noticeable gap in their knowledge and awareness of health initiatives related explicitly to NCDs. Some participants were aware of the role of government health programs like Ayushman Bharat in increasing public awareness. Still, none of them were aware of the (RBSK) and other health programs. This highlights the necessity for awareness and utilization of national programs.

While most participants were aware of the harms of tobacco use, their knowledge of the components of a healthy diet and the importance of regular physical activity was comparatively limited. Low knowledge can be attributed to unfavourable behaviors such as not reading nutrition labels, eating packaged foods, and engaging in sedentary activities, consistent with Bassi et al.‘s study, 2021 [24]. Participants mentioned a healthy lifestyle, such as having a daily routine, waking up early, and eating healthy food. This highlights educational institutions’ crucial role in fostering children’s health behaviors. Teachers, in particular, have played a significant role in promoting these practices, reflecting the integral part schools play in public health education.

Various education programs in India have been designed for school-going adolescents, but few comprehensive programs address diet, physical activity, and tobacco use, aligning with Saraf et al.’s study, 2015 [25]. Children may already have some understanding of the importance of physical activity but require interventions to create healthier environments that support this behaviour [26].

The thematic analysis identified several barriers to adopting healthy lifestyles. Accessibility barriers, such as limited recreational spaces and the prevalence of unhealthy food options, emerged as significant obstacles. Behavioural barriers include resistance to adopting healthy habits and preferences for unhealthy foods (taste preference). Advertisements also significantly influence dietary choices, reflecting broader societal challenges.

Academic and household responsibilities also restrict students’ opportunities for physical activity, highlighting a gap between knowledge and practice that necessitates targeted interventions. The easy availability of junk food products to maintain healthy behaviours related to diet and tobacco use, which echoes with Gupte et al.’s study, 2022 [27]. Socio-economic factors, such as the cost of healthy foods and financial constraints, exacerbate these challenges, underscoring the need for policies to improve access to nutritious options. As per Mathur et al.’s study 2021 [28], India is undergoing a rapid nutrition transition, with a growing preference for packaged, processed, and hyper palatable foods over traditional, nutritious diets or home-cooked meals, which is a major contributor to his shift. According to the WHO, a sedentary lifestyle and inadequate physical activity increase the risk of NCDs.

Parental attitudes also play a role in children’s dietary choices. Some parents prioritize convenience over health and resist dietary changes. Despite this, the study highlights the positive impact of family and peer support on encouraging healthy behaviours. Schools also contribute significantly by promoting health education and physical activities, supporting a conducive environment for healthy living. The pervasive influence of social media and movies on tobacco use and fast-food consumption among teenagers and youth adds another layer of complexity.

Family and peer support were identified as key facilitators of healthy behaviours, emphasizing the role of social support in adolescent health. Positive reinforcement from family and peers can significantly influence children’s health behaviours. As mentioned by the teachers, 80% of students are greatly impacted by peer influence. This highlights the importance of involving these groups in health promotion efforts. School initiatives, including strict anti-tobacco policies and promotion of physical activities, were also found to be effective strategies. The Government of India’s flagship initiatives, like the mid-day meal programme [29], Eat Right India [30], have been implemented nationwide to improve the nutritional outcomes in children and adolescents, advocating for school-based programs to foster a healthy student environment. The study’s endorsement of the WHO ‘Let’s Be Active’ campaign [31] reflects how participating schools recognized and supported the campaign’s goals by actively encouraging physical activity and integrating its health messages into school education and activities, as identified through interviews and focus groups.

## Recommendations

The primary challenge faced by India is the efficient implementation of the numerous prevention and control measures introduced by the government. To address this, it is essential to strengthen the health system to enhance access to clinical services, robust surveillance, and health promotion across various contexts [32]. Many participants in the study were unaware of several government programs such as RBSK, NPNCDs, despite some awareness of Ayushmann Bharat. Possible actions to curb this situation could be put together by school health committees, in collaboration with district health authorities by organising at least 2 information session per year to introduce and explain all government programmes. Comprehensive strategies that involve multiple stakeholders are critical to bridging the gap between knowledge and practice. It is recommended that there be enhanced and continuous communication and outreach to increase awareness of lesser-publicized health programs [33]. According to a study by Thakur et al., 2020 [32], a weak and fragmented health system hampers the ability to reach disadvantaged and marginalized populations. Future efforts should emphasize regular health monitoring, inclusive sports programs and enhanced health screenings. Given limited understanding of healthy diet, strong preference and easy availability of packaged/junk food, a practical step would be to place simple age-appropriate IEC materials in classrooms and canteen. Short, practical training modules for canteen staff, parents and teachers on healthy menu planning and portion size can be developed and shared along with monthly ‘healthy tiffin days’ designed to introduce healthy eating visible and aspirational. Limited recreational spaces in some areas, and academic load reduce time for physical activity. Based on these findings, cluster level school sports events and low-cost activities (e.g., local games, skipping, fast walking) are recommended, that can be organised so that schools with limited infrastructure can participate. Schools should keep 30 minutes of daily physical activity time (including but not limited to PT, yoga, mind-eye relaxation exercises) within the schedule specially for higher grade student. Peer influence and parental guidance are key gatekeepers of healthy diet and routines- with this as one of the finding regular parent-teacher meeting to share guidance on simple points such as – healthy tiffin, encouraging physical activites, limit screen time can be held. During these meetings family level health challenges (e.g., family walks, week of no sugar/green vegetables) can be proposed through schools to build motivation and enhance family support to healthy living. Community support through accessible recreational spaces and parental involvement in promoting healthy eating is vital.

Policymakers should prioritize improving access to recreational spaces and regulating the availability of unhealthy foods near schools. Behavioural interventions should focus on making healthy foods more appealing by addressing taste preferences and advertising. Creative strategies by parents and teachers are also recommended to improve children’s nutritional intake. Economic policies could play a significant role by lowering the cost of healthy foods and supporting health-promoting activities. Policy enforcement, diverse physical activities, and structured dietary plans are essential to creating an environment that promotes healthy lifestyles.

Engaging families and peers in health promotion and strengthening school-based programs will help cultivate an environment conducive to healthy behaviours. Informed and active parents can reinforce healthy habits at home, while collaborative efforts from schools and communities will significantly benefit children’s well-being. Recognising the importance of community action and advocacy highlights the need for policy and environmental changes. Further, developing context-specific intervention materials involving parents and communities is important to enhance health education activities. The Comprehensive School Health Program (CSHP) [34] should be carefully designed to ensure sufficient time is allocated for health promotion and NCD risk prevention. Developing intervention materials that can be embedded into the curriculum, with support from teacher and student ambassadors, is recommended for effective program implementation.

### Limitations

This study captures rich, firsthand qualitative insights from students, parents, teachers, and canteen staff across both rural and urban schools in Jodhpur using FGDs and in-depth interviews. However, only two canteen staff members were available and consented to participate, which limited the diversity of food-service perspectives. Additionally, restricted school hours and schedules further reduced participant diversity.

## Conclusions

Bridging the gap between health knowledge and practice requires a holistic approach among adolescents in Jodhpur. This requires concrete and specific age-appropriate context and measures. Enhancing communication about health programs by integrating information on NCDs and programs like RBSK, Ayushmann Bharat and NPNCD into routine classroom and parent-teacher activities. Improving access to recreational spaces and safeguarding daily time of physical activities in school while also implementing policies to regulate unhealthy foods are essential. Peer and family influence can be leveraged through parent focused activity counselling modules and peer led health clubs that address diet, physical activity, and tobacco use. Engaging families and educators and integrating health education into school curriculums will help promote healthier behaviours ingrain in adolescents’ lived experiences and reduce the risk of non-communicable diseases. By linking these insights directly to intervention design, stakeholder mobilization, and policy recommendations, this study will help develop sustainable strategies to contribute to long term reduction of NCD risk factors in school and similar settings.
