# Supplementary material for: Health behaviours and lifestyle challenges among school children: A qualitative research from Rajasthan, India
Source: PLoS One. 2026 Jul 7;21(7):e0351408. doi: 10.1371/journal.pone.0351408 (PMC13340814; doi:10.1371/journal.pone.0351408)
Supplement: Attachment — Submitted filename: Qualitative Thematic Paper - Response to Reviewers.docx [file pone.0351408.s001.docx]

**Response to Reviewers comments**

We are grateful to the Academic Editor and reviewers for their constructive feedback. We have revised the manuscript accordingly.

1. Clarify Objectives: Rewrite the Introduction to present the study objectives more clearly and precisely.

**Response** - Thank you for this helpful suggestion. We have revised the Introduction to sharpen and clarify the study objectives. The revised text under the introduction section now specifies that the study aims (i) to explore school children’s lifestyle habits related to diet, physical activity and tobacco use, and (ii) to identify barriers and facilitators to adopting a healthy lifestyle from the perspectives of students, teachers, parents, and canteen staff in rural and urban schools of Jodhpur district. (Pg. - 3)

2. Expand Methodology: In the Methods section, describe how participants were selected, explain why you chose FGDs versus IDIs for each stakeholder group, and detail how (and when) you determined that data saturation was reached.

**Response-**  Thank you for the comment. The Methods section has been expanded and clarified in three ways as mentioned. The participation selection was purposive and has been explained under the section -Study site and Participants in the revised manuscript (Pg. - 4).Choice of FGD and IDIs among participants were based on the availability of the parents, teachers and canteen staff indulging to their busy schedule and to get the depth of their personal experience. For students FGDs and IDIs were conducted to encourage interaction and get active involvement without fear or shyness. This has been included under the section - Data Collection and Management (Pg. - 5). The saturation of Data was defined as the point when responses were repeated related to the topic of healthy lifestyle behaviour and practice among adolescents. This has been now updated on page no. 5 under the method section in the revised manuscript.

3. Enrich Results: Add relevant verbatim quotes from participants to support your findings. Explicitly discuss any notable differences or similarities between rural and urban school settings.

**Response** - Thank you for this suggestion.We now have revised the Result section to mention differences and similarities between rural and urban schools within the narrative of themes in the revised manuscript.

4. Sharpen Recommendations: Revise the Discussion or Conclusion to make policy recommendations more concrete and feasible. Suggest specific actions or interventions based on your findings.

**Response -** Thank you for the valuable comment. We agree with the comment and have updated the recommendation and conclusion section in the revised manuscript. (Pg. - 22, 23 & 24)

5. Address Minor Issues: Review the manuscript for language and style, standardise the citation format, and include a statement about ethics approval (with approval number and institution).

**Response-** Thank you f or mentioning these points. We have checked and cleared all possible mistakes of language/tense in the revised manuscript. The citations has been checked for the standardised style of APA to be followed. The Ethics statement is written after conclusion section identifying the ethics committee and ethical approval by the participants.
