# Supplementary material for: Health behaviours and lifestyle challenges among school children: A qualitative research from Rajasthan, India
Source: PLoS One. 2026 Jul 7;21(7):e0351408. doi: 10.1371/journal.pone.0351408 (PMC13340814; doi:10.1371/journal.pone.0351408)
Supplement: Peer review history

# Peer review: Decision Letter 0

**DOI:** 10.1371/journal.pone.0351408.r001

**Authors:** Praveen Suthar

31 Mar 2026

PONE-D-25-64859

Health Behaviors and Lifestyle Challenges among School Children: A Qualitative Research from Rajasthan, India

PLOS One

Dear Dr. Sangwan,

Thank you for submitting your manuscript to PLOS ONE. After careful consideration, we feel that it has merit but does not fully meet PLOS ONE’s publication criteria as it currently stands. Therefore, we invite you to submit a revised version of the manuscript that addresses the points raised during the review process.

Please submit your revised manuscript by May 15 2026 11:59PM. If you will need more time than this to complete your revisions, please reply to this message or contact the journal office at plosone@plos.org. When you're ready to submit your revision, log on to https://www.editorialmanager.com/pone/ and select the 'Submissions Needing Revision' folder to locate your manuscript file.

Please include the following items when submitting your revised manuscript:

- A letter that responds to each point raised by the academic editor and reviewer(s). You should upload this letter as a separate file labeled 'Response to Reviewers'.
- A marked-up copy of your manuscript that highlights changes made to the original version. You should upload this as a separate file labeled 'Revised Manuscript with Track Changes'.
- An unmarked version of your revised paper without tracked changes. You should upload this as a separate file labeled 'Manuscript'.

If you would like to make changes to your financial disclosure, please include your updated statement in your cover letter. Guidelines for resubmitting your figure files are available below the reviewer comments at the end of this letter.

If applicable, we recommend that you deposit your laboratory protocols in protocols.io to enhance the reproducibility of your results. Protocols.io assigns your protocol its own identifier (DOI) so that it can be cited independently in the future. For instructions see: https://journals.plos.org/plosone/s/submission-guidelines#loc-laboratory-protocols. Additionally, PLOS ONE offers an option for publishing peer-reviewed Lab Protocol articles, which describe protocols hosted on protocols.io. Read more information on sharing protocols at https://plos.org/protocols?utm_medium=editorial-email&utm_source=authorletters&utm_campaign=protocols.

As the corresponding author, your ORCID iD is verified in the submission system and will appear in the published article. PLOS supports the use of ORCID, and we encourage all coauthors to register for an ORCID iD and use it as well. Please encourage your coauthors to verify their ORCID iD within the submission system before final acceptance, as unverified ORCID iDs will not appear in the published article. *Only*  the individual author can complete the verification step; PLOS staff *cannot*  verify ORCID iDs on behalf of authors.

We look forward to receiving your revised manuscript.

Kind regards,

Praveen Suthar, PhD (Public Health), MPH

Academic Editor

PLOS One

Journal Requirements:

When submitting your revision, we need you to address these additional requirements.

1. Please ensure that your manuscript meets PLOS ONE's style requirements, including those for file naming. The PLOS ONE style templates can be found at

https://journals.plos.org/plosone/s/file?id=wjVg/PLOSOne_formatting_sample_main_body.pdf and

https://journals.plos.org/plosone/s/file?id=ba62/PLOSOne_formatting_sample_title_authors_affiliations.pdf

2. In your ethics statement, please ensure you have provided the full name of the ethics committee(s) that approved this study. If more than 5 committees provided approval, it is acceptable to provide a list as a Supporting Information file.

3. Thank you for stating the following financial disclosure:

“No. 79/1/IIRP-136/CVD/2023-NCD”

Please state what role the funders took in the study.  If the funders had no role, please state: "The funders had no role in study design, data collection and analysis, decision to publish, or preparation of the manuscript."

If this statement is not correct you must amend it as needed.

Please include this amended Role of Funder statement in your cover letter; we will change the online submission form on your behalf.

4. We note that your Data Availability Statement is currently as follows: [All relevant data are within the manuscript and its Supporting Information files.]

Please confirm at this time whether or not your submission contains all raw data required to replicate the results of your study. Authors must share the “minimal data set” for their submission. PLOS defines the minimal data set to consist of the data required to replicate all study findings reported in the article, as well as related metadata and methods (https://journals.plos.org/plosone/s/data-availability#loc-minimal-data-set-definition).

For example, authors should submit the following data:

- The values behind the means, standard deviations and other measures reported;

- The values used to build graphs;

- The points extracted from images for analysis.

Authors do not need to submit their entire data set if only a portion of the data was used in the reported study.

If your submission does not contain these data, please either upload them as Supporting Information files or deposit them to a stable, public repository and provide us with the relevant URLs, DOIs, or accession numbers. For a list of recommended repositories, please see https://journals.plos.org/plosone/s/recommended-repositories.

If there are ethical or legal restrictions on sharing a de-identified data set, please explain them in detail (e.g., data contain potentially sensitive information, data are owned by a third-party organization, etc.) and who has imposed them (e.g., an ethics committee). Please also provide contact information for a data access committee, ethics committee, or other institutional body to which data requests may be sent. If data are owned by a third party, please indicate how others may request data access.

5. If the reviewer comments include a recommendation to cite specific previously published works, please review and evaluate these publications to determine whether they are relevant and should be cited. There is no requirement to cite these works unless the editor has indicated otherwise.

Additional Editor Comments (if provided):

Dear Author,

Thank you for submitting your manuscript on lifestyle behaviors and barriers among schoolchildren in Rajasthan. The study addresses an important and timely public health issue and demonstrates strong potential, particularly with its qualitative approach and engagement of multiple stakeholders.

However, we kindly request you to revise the manuscript to further strengthen its clarity and rigor as follows:

1. Clarify Objectives: Rewrite the Introduction to present the study objectives more clearly and precisely.

2. Expand Methodology: In the Methods section, describe how participants were selected, explain why you chose FGDs versus IDIs for each stakeholder group, and detail how (and when) you determined that data saturation was reached.

3. Enrich Results: Add relevant verbatim quotes from participants to support your findings. Explicitly discuss any notable differences or similarities between rural and urban school settings.

4. Sharpen Recommendations: Revise the Discussion or Conclusion to make policy recommendations more concrete and feasible. Suggest specific actions or interventions based on your findings.

Address Minor Issues: Review the manuscript for language and style, standardize the citation format, and include a statement about ethics approval (with approval number and institution).

Regards

[Note: HTML markup is below. Please do not edit.]

Reviewers' comments:

Reviewer's Responses to Questions

**Comments to the Author**

1. Is the manuscript technically sound, and do the data support the conclusions?

The manuscript must describe a technically sound piece of scientific research with data that supports the conclusions. Experiments must have been conducted rigorously, with appropriate controls, replication, and sample sizes. The conclusions must be drawn appropriately based on the data presented.

Reviewer #1: Yes

**********

2. Has the statistical analysis been performed appropriately and rigorously?

Reviewer #1: Yes

**********

3. Have the authors made all data underlying the findings in their manuscript fully available?

The PLOS Data policy requires authors to make all data underlying the findings described in their manuscript fully available without restriction, with rare exception (please refer to the Data Availability Statement in the manuscript PDF file). The data should be provided as part of the manuscript or its supporting information, or deposited to a public repository. For example, in addition to summary statistics, the data points behind means, medians and variance measures should be available. If there are restrictions on publicly sharing data—e.g. participant privacy or use of data from a third party—those must be specified.

Reviewer #1: Yes

**********

4. Is the manuscript presented in an intelligible fashion and written in standard English?

PLOS ONE does not copyedit accepted manuscripts, so the language in submitted articles must be clear, correct, and unambiguous. Any typographical or grammatical errors should be corrected at revision, so please note any specific errors here.

Reviewer #1: Yes

**********

5. Review Comments to the Author

Please use the space provided to explain your answers to the questions above. You may also include additional comments for the author, including concerns about dual publication, research ethics, or publication ethics. (Please upload your review as an attachment if it exceeds 20,000 characters)

Reviewer #1: The manuscript explores an important issue of lifestyle behaviors and barriers among schoolchildren in Rajasthan, using qualitative methods with multiple stakeholders. It is timely and relevant, but the objectives need clearer articulation, and methodological details—such as participant selection, rationale for FGDs versus IDIs, and data saturation—should be expanded. Results would be stronger if illustrated with participant quotes and more nuanced rural–urban comparisons. Policy recommendations are valuable but should be more specific and actionable. Minor issues include language clarity, citation consistency, and inclusion of ethics approval details. Overall, the study has strong potential but requires revisions to improve methodological rigor, enrich findings, and sharpen recommendations.

**********

6. PLOS authors have the option to publish the peer review history of their article (what does this mean?). If published, this will include your full peer review and any attached files.

If you choose “no”, your identity will remain anonymous but your review may still be made public.

**Do you want your identity to be public for this peer review?**  For information about this choice, including consent withdrawal, please see our Privacy Policy.

Reviewer #1: No

**********

[NOTE: If reviewer comments were submitted as an attachment file, they will be attached to this email and accessible via the submission site. Please log into your account, locate the manuscript record, and check for the action link "View Attachments". If this link does not appear, there are no attachment files.]

To ensure your figures meet our technical requirements, please review our figure guidelines: https://journals.plos.org/plosone/s/figures

You may also use PLOS’s free figure tool, NAAS, to help you prepare publication quality figures: https://journals.plos.org/plosone/s/figures#loc-tools-for-figure-preparation.

NAAS will assess whether your figures meet our technical requirements by comparing each figure against our figure specifications.

# Author comment: Author response to Decision Letter 1

**DOI:** 10.1371/journal.pone.0351408.r002

24 May 2026

Editor's Comments

1. Please amend the title either on the online submission form or in your manuscript so that they are identical.

Response - Thank you for notifying us. We have amended the title on the online submission.

2. We note that your Data Availability Statement is currently as follows: [All relevant data are within the manuscript and its Supporting Information files.]

Response - Thank you for the comment. We have attached all the relevant data within the manuscript, therefore edited the Data Availability Statement accordingly.

Response to Reviewers comments

We are grateful to the Academic Editor and reviewers for their constructive feedback. We have revised the manuscript accordingly.

1. Clarify Objectives: Rewrite the Introduction to present the study objectives more clearly and precisely.

Response - Thank you for this helpful suggestion. We have revised the Introduction to sharpen and clarify the study objectives. The revised text under the introduction section now specifies that the study aims (i) to explore school children’s lifestyle habits related to diet, physical activity and tobacco use, and (ii) to identify barriers and facilitators to adopting a healthy lifestyle from the perspectives of students, teachers, parents, and canteen staff in rural and urban schools of Jodhpur district. (Pg. - 3)

2. Expand Methodology: In the Methods section, describe how participants were selected, explain why you chose FGDs versus IDIs for each stakeholder group, and detail how (and when) you determined that data saturation was reached.

Response- Thank you for the comment. The Methods section has been expanded and clarified in three ways as mentioned. The participation selection was purposive and has been explained under the section -Study site and Participants in the revised manuscript (Pg. - 4).Choice of FGD and IDIs among participants were based on the availability of the parents, teachers and canteen staff indulging to their busy schedule and to get the depth of their personal experience. For students FGDs and IDIs were conducted to encourage interaction and get active involvement without fear or shyness. This has been included under the section - Data Collection and Management (Pg. - 5). The saturation of Data was defined as the point when responses were repeated related to the topic of healthy lifestyle behaviour and practice among adolescents. This has been now updated on page no. 5 under the method section in the revised manuscript.

3. Enrich Results: Add relevant verbatim quotes from participants to support your findings. Explicitly discuss any notable differences or similarities between rural and urban school settings.

Response - Thank you for this suggestion.We now have revised the Result section to mention differences and similarities between rural and urban schools within the narrative of themes in the revised manuscript.

4. Sharpen Recommendations: Revise the Discussion or Conclusion to make policy recommendations more concrete and feasible. Suggest specific actions or interventions based on your findings.

Response - Thank you for the valuable comment. We agree with the comment and have updated the recommendation and conclusion section in the revised manuscript. (Pg. - 22, 23 & 24)

5. Address Minor Issues: Review the manuscript for language and style, standardise the citation format, and include a statement about ethics approval (with approval number and institution).

Response- Thank you for mentioning these points. We have checked and cleared all possible mistakes of language/tense in the revised manuscript. The citations has been checked for the standardised style of APA to be followed. The Ethics statement is written after conclusion section identifying the ethics committee and ethical approval by the participants.

Attachment Submitted filename: Qualitative Thematic Paper - Response to Reviewers.docx

# Editor report: Decision Letter 1

**DOI:** 10.1371/journal.pone.0351408.r003

**Authors:** Praveen Suthar

27 May 2026

Health Behaviours and Lifestyle Challenges among School Children: A Qualitative Research from Rajasthan, India

PONE-D-25-64859R1

Dear Dr. Sangwan,

We’re pleased to inform you that your manuscript has been judged scientifically suitable for publication and will be formally accepted for publication once it meets all outstanding technical requirements.

Within one week, you’ll receive an e-mail detailing the required amendments. When these have been addressed, you’ll receive a formal acceptance letter and your manuscript will be scheduled for publication.

An invoice will be generated when your article is formally accepted. Please note, if your institution has a publishing partnership with PLOS and your article meets the relevant criteria, all or part of your publication costs will be covered. Please make sure your user information is up-to-date by logging into Editorial Manager at Editorial Manager® and clicking the ‘Update My Information' link at the top of the page. For questions related to billing, please contact billing support.

If your institution or institutions have a press office, please notify them about your upcoming paper to help maximize its impact. If they’ll be preparing press materials, please inform our press team as soon as possible -- no later than 48 hours after receiving the formal acceptance. Your manuscript will remain under strict press embargo until 2 pm Eastern Time on the date of publication. For more information, please contact onepress@plos.org.

Kind regards,

Praveen Suthar, PhD (Public Health), MPH

Academic Editor

PLOS One

Additional Editor Comments (optional):

Reviewers' comments:

# Editor report: Acceptance letter

**DOI:** 10.1371/journal.pone.0351408.r004

**Authors:** Praveen Suthar

PONE-D-25-64859R1

PLOS One

Dear Dr. Sangwan,

I'm pleased to inform you that your manuscript has been deemed suitable for publication in PLOS One. Congratulations! Your manuscript is now being handed over to our production team.

At this stage, our production department will prepare your paper for publication. This includes ensuring the following:

* All references, tables, and figures are properly cited

* All relevant supporting information is included in the manuscript submission,

* There are no issues that prevent the paper from being properly typeset

You will receive further instructions from the production team, including instructions on how to review your proof when it is ready. Please keep in mind that we are working through a large volume of accepted articles, so please give us a few days to review your paper and let you know the next and final steps.

Lastly, if your institution or institutions have a press office, please let them know about your upcoming paper now to help maximize its impact. If they'll be preparing press materials, please inform our press team within the next 48 hours. Your manuscript will remain under strict press embargo until 2 pm Eastern Time on the date of publication. For more information, please contact onepress@plos.org.

You will receive an invoice from PLOS for your publication fee after your manuscript has reached the completed accept phase. If you receive an email requesting payment before acceptance or for any other service, this may be a phishing scheme. Learn how to identify phishing emails and protect your accounts at https://explore.plos.org/phishing.

If we can help with anything else, please email us at customercare@plos.org.

Thank you for submitting your work to PLOS ONE and supporting open access.

Kind regards,

PLOS ONE Editorial Office Staff

on behalf of

Dr. Praveen Suthar

Academic Editor

PLOS One
